# Antimutagenic, antigenotoxic and antiproliferative activities of *Fraxinus angustifolia* Vahl. leaves and stem bark extracts and their phytochemical composition

**DOI:** 10.1371/journal.pone.0230690

**Published:** 2020-04-16

**Authors:** Ghania Bouguellid, Chiara Russo, Margherita Lavorgna, Concetta Piscitelli, Karima Ayouni, Erica Wilson, Hye Kyonn Kim, Rob Verpoorte, Young Hae Choi, Dina Kilani-Atmani, Djebbar Atmani, Marina Isidori

**Affiliations:** 1 Laboratoire de Biochimie Appliquée, Faculté des Sciences de la Nature et de la Vie, Université de Bejaia, Bejaia, Algeria; 2 Dipartimento di Scienze e Tecnologie Ambientali, Biologiche e Farmaceutiche, Università della Campania “Luigi Vanvitelli”, Caserta, Italy; 3 Natural Products Laboratory, Institute of Biology, Leiden University, Leiden, The Netherlands; Duke University School of Medicine, UNITED STATES

## Abstract

In recent years, chronic degenerative diseases such as certain types of cancers, are becoming an evident issue. DNA damage has been for long recognized as a causal factor for cancer development because mutations or chromosomal aberrations affect oncogenes and tumor suppressor genes leading cells to malignant transformation and to the subsequent cancerous growth. Medicinal plants are often used for the prevention or treatment of various diseases with great scientific interest. Among the medicinal plants distributed in the Mediterranean region, *Fraxinus angustifolia* Vahl. has been used in traditional medicine for its remarkable curative properties. However, in spite of this popularity, little works have been performed on the activity so that further studies should be performed to investigate in depth the antimutagenic, antigenotoxic and antiproliferative activities of the plant. Thus, the present study was aimed to the evaluation of the potential antimutagenic, antigenotoxic and antiproliferative properties of leaves and stem bark extracts of this well-known tree. Antimutagenic activity was evaluated by Salmonella mutagenicity assay in *Salmonella typhimurium* TA98 and TA100 strains. The antigenotoxic potential was assessed by *umu* test in the strain of *S*. *typhimurium* TA1535/pSK1002. Antiproliferative activity was studied on human hepatoblastoma (HepG-2) and on breast adenocarcinoma (MCF-7) cell lines by MTT assay. Furthermore, the antiproliferative activity observed on cancer cells was compared with that on the human normal-like fibroblasts (TelCOFS02MA) and the selectivity index was calculated to understand if extracts were able to exert selective toxicity towards cancer cells. Moreover, phenolic compounds are plant substances with a large spectrum of biochemical activities with antioxidant, antimutagenic and anticarcinogenic effects. Based on the strong evidence of biological activities of phenolic compounds, the study was focused on the determination of total phenolics and flavonoids contents, and the phytochemical composition of the extracts assessed by LC/MS. The ethanol extracts of both leaves and stem barks showed significant from moderate to strong antimutagenic and antigenotoxic effects. In addition, selective cytotoxicity towards cancer cells was shown by ethanolic leaves extract and aqueous/chloroform leaves and stem bark extracts. The latter showed high levels of total phenolic contents among all the other extracts. Identified phenylethanoids (calceolariosides, verbascoside) and secoiridoids (oleuropein and ligstroside) could be responsible for the demonstrated broad spectrum of healthy properties.

## Introduction

Natural products still play a leading role in the treatment of various diseases in diverse forms e.g. extracts, fractions or as a chemical platform. In the history of humanity, plants have always been the most popular source of medicines, mainly thanks to their secondary metabolites with many pharmacological properties. The knowledge of the various healing properties of plants has been transmitted primarily in an empirical way based on folk traditions, and then validated with scientific evidences [1]. At present, one of the hottest topic in medicine is focused on natural bioactive products in the prevention and/or treatment of chronic diseases which have been characterized as the public health challenge of the 21^st^ century. In fact, as explained by Lunenfeld and Stratton [2], in developed countries the rise in healthcare systems and life expectancy, as well as the decrease in fertility rate mainly due to chromosomal abnormalities lead to a rapid increase in the world population aging with consequent chronic degenerative disease increase. In line with the World Health Organization (WHO, 2018), cancer is the second chronic disease (9.6 million deaths in 2018), and cancers of liver (782 000 deaths) and breast (627 000 deaths) are among the most common causes of cancer death. As reported [3], the DNA damage has been long recognized as causal factor for cancer development. In fact, mutations or chromosomal aberrations affect oncogenes and tumor suppressor genes leading cells to malignant transformation. Hence, antimutagenic, antigenotoxic and anticarcinogenic substances play a major role in the primary prevention of cancer development [4, 5].

In recent years, a wide range of medicinal plants and their metabolites have been studied for their potential to decrease the mutagenic and carcinogenic effects of potentially damaging chemicals [6, 7]; in fact, these natural compounds are able to inhibit free radicals and oxidative stress-induced DNA and cellular damages [8, 9].

One of medicinal plants widely distributed in the Mediterranean region is *Fraxinus angustifolia* Vahl a medium-sized deciduous tree, belonging to the Oleaceae family. The plant is extensively used in traditional medicine for its remarkable curative properties. In Algerian folk medicine, different parts of this plant are used to treat many inflammatory diseases [10]; leaves and fruits (samaras) are used in decoctions and infusions as anti-rheumatism, while bark is effective for curing hemorrhoids and fever [11]. Moreover, a mixture composed of powder of samaras, olive oil and honey is used against gonorrhea [12]. Moreover, an exudate from the bark of *F*. *angustifolia Vahl*. or *F*.*ornus L*., called manna, collected in Southern Italy, is used in case of digestive problems [13] as well as expectorant and sedative in cough [14, 15]. When taken in hypertonic solutions, manna acts as a dehydrating agent in the treatment of wounds, ulcers and promotes the flow of the contents of the gall bladder and bile ducts [16, 17].

According to European Medicines Agency (EMA) [18], the biological activities of *F*. *angustifolia* Vahl. in traditional applications is attributed to the phytochemical constituents; e.g. flavonoids, coumarins, iridoids and secoiridoids having anti-inflammatory and analgesic effects (laxative and diuretic effects might be associated with to mannitol). Recently, based on the chemical structure study [19], phenylethanoids such as calceolariosides (A and B) and verbascoside are the major antioxidants of stem bark extracts of *F*. *angustifolia* Vahl. Furthermore, verbascoside was also identified in leaves fractions, in addition to secoiridoids (oleuropein and ligstroside) and at least three flavonoides glycosides: kaempferol 3-*O*-rutinoside, isoquercetrin (quercetin 3-*O*-glucoside) and rutin (quercetin 3-*O*-α-L-rhamnopyranosyl-1-6-glucopyranose) which were responsible for leaves antioxidant potential. Furthermore, the latter could also be behind the anti-enzymatic [20–22], antidiabetic, and hepatoprotective [23], as well as wound healing properties [24].

However, to the best of our knowledge, in spite of this popularity, the antimutagenic, antigenotoxic and antiproliferative activities of *F*. *angustifolia* Vahl leaves and stem bark extracts had never been tested. In this context, the present study aimed to evaluate the antimutagenic, antigenotoxic and antiproliferative properties of *F*. *angustifolia* Vahl. leaves and stem bark extracts, and to extend the knowledge on their phytochemical composition.

The antimutagenic activity was evaluated by the *Salmonella* mutagenicity assay (Ames test) in *Salmonella typhimurium* TA98 and TA100 strains, auxotrophs for histidine (His^-^). Furthermore, the antigenotoxic potential was assessed by *umu*-test in *S*. *typhimurium* TA1535/pSK1002. Both antimutagenic and antigenotoxic assays were performed with and without liver homogenate (S9) to find out if metabolizing enzymes were involved in the activation of these natural compounds. Obviously, before beginning antimutagenic and antigenotoxic assays, Ames and *umu* tests were performed also to assess the eventual mutagenicity and genotoxicity of *F*. *angustifolia* Vahl. extracts.

Antitumor potency was studied by testing *F*. *angustifolia* Vahl. leaves and stem bark extracts on human hepatoblastoma (HepG-2) and on breast adenocarcinoma (MCF-7) cell lines (two among the most widespread cancers) by MTT assay. Then, to understand if extracts were able to exert selective toxicity towards cancer cells, the antiproliferative activity observed on cancer cells was compared with that of the human normal-like fibroblast cell line (TelCOFS02MA).

Moreover. In order to study the chemical basis of the results obtained, the polyphenolic composition of most active extracts of *F*. *angustifolia* Vahl. were characterised after fractionation using HPLC/MS method.

## Materials and methods

### Reagents

Ethanol (CAS: 64-17-5), Ethyl acetate (CAS: 141-78-6) and Chloroform (CAS: 67-66-3) were purchased from Prolabo (Sion, Switzerland). Aroclor 1254-induced male Sprague Dawley rat liver (S9) was purchased from TrinovaBiochem GmbH (Giessen, Germany). Sodium azide (NaN_3_, CAS: 26628-22-8) was from JT Baker (Milano, Italy). 2-Nitrofluorene (2-NF, CAS: 607-57-8), 2-amino-anthracene (2-AA, CAS: 613-13-8), 3-methylcholanthrene (3-MC, CAS: 56-49-5), 4-nitroquinoline-1-oxide (4-NQO, CAS: 56-57-5), Cyclophosphamide (CP, CAS: 6055-19-2), L-histidine (CAS: 71-00-1), Biotin (CAS: 58-85-5), and *O*-Nitrophenyl-β-D-galactopyranoside (ONPG, CAS: 369-07-3), 3-(4,5-dimethylthiazol-2-yl)-2,5diphenyl-tetrazolium bromide (MTT, CAS: 298-93-1) were purchased from Sigma-Aldrich (Milano, Italy). HEPES and Roswell Park Memorial Institute medium (RPMI 1640) were supplied by Lonza BioWhittaker (Verviers, Belgium).

### Sample collection and extraction procedure

*Fraxinus angustifolia* Vahl. leaves (FL) and stem bark (FB) were collected in July 2014 in remote areas of Chemini (963 m altitude, 36°35′ latitude and 4°36′ longitude), located in the department of Bejaia (Northeastern Algeria). *F*. *angustifolia* Vahl is not on the list of protected species, therefore no permission is required to collect it according to Algerian legislation [Art. 4 n°12–03 of 4/01/2012]. A voucher specimen, with the number (O/n°59), taxonomically validated by Professor Hacène Abdelkrim. was deposited in the herbarium of Department of Botany, ENSA (Ecole Nationale des Sciences Agronomiques), El-Harrach (Algiers, Algeria).

Leaves and stem bark samples were shade dried at room temperature, then ground into fine powders using an electric blender (KIKA, Labortechnic,Staufen, Germany). Phenolic compounds were extracted following the procedure described [25] and modified [26]. An amount of 100 g of each plant material was macerated in ethanol 96% (1:4 w/v) for 24 h under continuous stirring. After decantation, supernatants were dried using a rotavapor (HEIDOLF). The crude ethanolic extracts (FL1 and FB1, for leaves and stem bark, respectively) were further partitioned using ethyl acetate and distilled water (1:3:1/w:v:v) leading to ethyl acetate fractions (FL2 and FB2) and their respective aqueous fractions (FL3 and FB3). The FL2 and FB2 fractions were then partitioned between chloroform and distilled water (1:3:1/w:v:v) in order to obtain chloroform (FL4 and FB4) and aqueous (FL5 and FB5) fractions.

### Salmonella mutagenicity assay (Ames test)

The mutagenic and antimutagenic activities of *F*. *angustifolia Vahl*. extracts were evaluated by the *Salmonella*/microsome assay, using TA98 and TA100 *S*. *typhimurium* strains [27, 28]. Salmonella strains were from the permanent collection of the Laboratory of Hygiene and Environmental Toxicology, University of Campania, Italy. TA98 strain was used to observe frame-shift mutations, while TA100 was used to assess base-pair substitutions due to missense mutations. Briefly, 100 μL of *S*. *typhimurium*TA98 or TA100 cultured overnight (10^8^ cells), 100 μL of extract at 1000 μg/mL, chosen as the highest concentration for genotoxicity assessment, and 500 μL of phosphate buffer (0.1 M, pH = 7.4), or S9 mixture (lyophilized S9, 1 M NADP, 1 M Glucose-6-phosphate, 0.1 M Phosphate buffer pH 7.4, 0.4 M MgCl_2_, 0.4 M KCl) in case of metabolic activation, were added to 2.5 mL of 0.5 M histidine-biotin top agar, and then poured onto minimal glucose agar, in triplicate. Furthermore, 2-NF at 2.5 and 5 μg/mL for TA98, and NaN_3_ at 5 and 10 μg/mL for TA100 were used as direct positive controls in absence of metabolic activation. Moreover, 25 and 50 μg/mL of 3-MC for TA98, 50 and 100 μg/mL of CP for TA100 were used as indirect positive controls in presence of metabolic activation. saline solution (0.9% NaCl) was used as negative control. After incubation (37°C/72 h), induced His^+^ revertants were counted and statistically compared to the number of spontaneous revertants on the negative control plate. Plates count after 72h instead of 48h was chosen to facilitate the reading.

The mutagenic ratio (MR) was calculated for each tested extract as the ratio between the average number of revertants per plate of the test compound and the average number of spontaneous revertants per plate of the negative control. A sample was considered mutagenic when a two-fold increase in the number of mutants (MR ≥ 2) was observed.

For the antimutagenic assay, three different concentrations of the extracts (25, 50 and 100 μg/mL, chosen starting dilutions from the highest concentration of the value 1/10 lower than the concentration used to evaluate the eventual mutagenic effect, 1000 μg/mL) were pre-incubated with direct and indirect mutagens for 2 h at 37°C. The results were expressed as the percentage of the ability of the extracts to inhibit the action of the mutagen, calculated as follows:

$$Inhibition\;(\%)=100-\left[(\frac{T}{M})∙100\right]$$

Where T is the mean number of revertant colonies in the plate containing both mutagen and test compounds, M is the mean number of revertant colonies in the plate containing only the mutagen [29].

No antimutagenic effect was recorded when inhibition was lower than 25%, a moderate effect for an inhibition value between 25% and 40%, and strong antimutagenicity for values greater than 40% [29].

### Umu test

The SOS/*umu* bioassay detects the induction of the SOS-repair system in the *S*. *typhimurium* TA1535/pSK1002 strain, whose plasmidcarries the *umuC*:*lacZ* fusion gene with the β-galactosidase activity strictly dependent on *umuC* expression in response to specific DNA damaging agents [30]. The *S*. *typhimurium* TA1535/pSK1002 (optical density of ≥ 800 Formazine Nephelometric Units, FNU) was purchased by EBPI (Burlington, Ontario, Canada) and cultured in tryptone, glucose and ampicillin (TGA)-culture medium at 37°C for 12 h, and then was ten-fold diluted. After 2 h-reincubation, 70 μL of exponentially growing bacteria (340–350 FNU) were mixed with 180 μL of the respective extract (1000 μg/mL), chosen as the highest concentration for genotoxicity assessment, and 20 μL TGA medium (ten-fold concentrated) into each well of the 96-well microplate, in triplicates. Moreover, saline solution (0.9% NaCl) was used as negative control, 4-NQO (0.05 μg/mL) and 2-AA (0.2 μg/mL) were used as positive controls in the absence and presence of S9, respectively. The blank contained 70 μL of TGA medium instead of the 70 μL of the bacterial culture. For the determination of indirect genotoxins, 450 μL of S9 mix (lyophilized S9, 1 M NADP and 1 M Glucose-6-phosphate) were added to 15 mL of bacterial medium. After incubation for 2 h at 37°C under shaking at 150 rpm, 30 μl from each well were transferred in a new microplate containing fresh medium and re-incubated for a second time. The density of the strain was monitored by measuring the absorbance at 620 nm using a microplate reader (TecanSPECTRAfluor, Männedorf, Switzerland).

For the determination of β-galactosidase activity, 30 μL of TGA medium was mixed with 120 μL of B-buffer (0.1 M sodium phosphate pH 7.4, 10 mM KCl, 1 mM MgSO_4_, 1 mg/L β -mercaptoethanol, 10 μL of SDS 1 mg/mL) and the enzymatic reaction was activated by adding 30 μL of 4.5 mg/mL ONPG. After 30 min of incubation at 28°C, 120 μL Na_2_CO_3_ (1M) were added to stop the reaction. The absorbance was recorded at 420 nm.

Genotoxicity was quantified by induction ratio (IR) which is the ratio of absorbance at 420 nm of the sample (T) and of the negative control (N), corrected for growth rate at 620 nm. The sample is considered genotoxic when the induction ratio (IR) is equal to or higher than 1.5, calculated as the ß-galactosidase activity of the sample relatively to the negative control:

$$IR=\frac{{ßgal.units}_{T}}{{ßgal.units}_{N}}$$

The β-galactosidase activity (βgal. unit) was calculated as follows:

$$\beta gal.unit=\frac{\left(A_{420T}-A_{420B}\right)}{\left(A_{620T}-A_{620B}\right)}$$

Where: A_420_ is the absorbance at 420 nm relative to enzymatic reaction intensity of samples (T) and blank (B); while A_620_ is the absorbance at 620 nm of bacteria growth of samples and blank.

The antigenotoxicity assay was conducted using the same procedure of the genotoxicity assay described above. Three different concentrations of extracts (25, 50 and 100 μg/mL, chosen starting dilutions from the highest concentration of the value 1/10 lower than the concentration used to evaluate the eventual genotoxic effect, 1000 μg/mL) were pre-incubated for 2 h at 37°C with known genotoxins, 4-NQO and 2-AA in the absence and presence of metabolic activation S9, respectively. The percentage of antimutagenicity was calculated [31]:

$$\%Antimutagenicity=[1‐(\beta gal\;{\mathrm{unit}}_{\mathrm{GENOTOXIN}+\mathrm{SAMPLE}}/\beta gal\;{\mathrm{unit}}_{\mathrm{GENOTOXIN}})]\cdot 100$$

The extract was considered as a strong antigenotoxic when its activity is above 70%, medium when it’s between 40 and 70% and neutral when lower than 40% [32].

### MTT assay

The antiproliferative activity of *F*. *angustifolia* Vahl. leaves and stem bark extracts was performed using the tetrazolium dye colorimetric test (MTT assay) [33] on Hep-G2 and MCF-7 cell lines. The cells used were provided by Prof. Abbondanza, Department of Precision Medicine, University of Campania. Periodic quality control testing procedures (authentication, characterization and mycoplasma testing) were performed. The test is based on the activity of mitochondrial reductases that convert tetrazolium salts into formazan, obtaining a purple solution in living cells. Briefly, cells were grown in RPMI1640 medium with 10% fetal bovine serum (FBS), 2% HEPES, 2% L-Glutamine, and 1% penicillin/streptomycin (10,000 U/mL), and were cultured in T-75-cm^2^ tissue culture flasks in humidified atmosphere of 95% air plus 5% CO_2_ at 37°C. When the cells were at 80%–90% of confluence; they were collected and counted with the vital dye trypan blue. The cells (1×10^4^/well) were seeded in quadruplicate wells of 96-well plates. After 24 h incubation at 37°C, the medium was removed and replaced with 200 μL of different concentrations **(**50 to 2000 μg/mL, chosen after range finding tests) of *F*. *angustifolia* Vahl. leaves and stem bark extracts dissolved in RPMI, chosen after range finding tests. The plates were then incubated for 72 h at 37°C. Each plate had negative control wells containing only the medium. After that, 20 μL of yellow MTT solution (5 mg/mL) was added to each well and the plates were re-incubated for 4 h at 37°C. Hence, the culture medium was removed and 200 μL /well of 2-propanol was used for dissolving the formazan. The absorbance of formazan was measured spectrophotometrically at 590 nm. The results were reported as inhibition cell (IC) percentage:

$$IC(\%)=1-\left[\frac{{OD}_{590}\;sample}{{OD}_{590}\;negative\;control}\right].100$$

Independent experiments were pooled and statistically analysed by Prism 5.03 version (GraphPad Inc., San Diego, CA, USA). Thus, IC_50_ values were calculated by non-linear concentration/response regression model.

Furthermore, selectivity index (SI) was calculated [34] to compare the antiproliferative activity observed on cancer cells with that of the human normal-like fibroblast cell line (TelCOFS02MA, ATCC® CRL 4005^™^ purchased from American Type Culture Collection, Milan, Italy). A selective toxicity towards cancer cells occurs when SI value is greater than 2. SI value was calculated using the formula given below:

$$SI=\frac{{IC}_{50}normal\;like\;cells}{{IC}_{50}\;cancer\;cells}$$

### Determination of total phenolics and flavonoids contents

The total phenolics contents of *F*. *angustifolia* Vahl. extracts were quantified by the Folin-Ciocalteu method and the amounts of phenolics were calculated from catechin standard curve [35]. Flavonoids contents were determined by the aluminium chloride procedure [36] and deduced from a rutin standard curve to be expressed as μg of catechin equivalent/mg of dry extract.

### UHPLC/MS investigation

The LC-MS analyses were carried out on the apparatus consisting of the following elements: Dionex Ultimate 3000 UHPLC system equipped with a quaternary pump (RS pump), an autosampler (RS autosampler) and UV detector with diode arrays (PE 785 A) linked to a high-resolution triple-quadrupole mass spectrometer (Brucker micro OTOF-QII, Bremen, Germany), equipped with an electrospray ionisation interface. The ethanolic extracts and their respective fractions of leaves and stem barks of *F*. *angustifolia* Vahl. (1 mg/mL) were injected onto a C18 Symetry HPLC column (Waters) (3.5 μm, 75 x 4.6 mm). The separations were carried out at room temperature with a mobile phase consisting of two water-acid solvents. Formic acid 0.1% (A) and methanol-0.1% formic acid (B) under the following conditions: 0 to 15 min, 13% B for 15 min, then in 5 min a linear gradient of 55 to 85% B then 85% B for 1 min, finally, return to the initial conditions (15% B) in 2 minutes to rebalance the column before any new injection. For all analyses, the solvents used are of HPLC quality, the flow rate was set at 0.350 mL/min, and the measurement wavelengths were set at 280, 360 and 450 nm. For comparative purposes, the injection volume and the concentrations of the injected solutions were fixed at 5 μL and 1 mg/mL, respectively, for all injected extracts and fractions.

### Statistical analysis

Tests were from three independent experiments. The results were expressed as mean and standard deviation. Analysis of variance (one-way ANOVA model) was performed in combination with *Dunnett’s post-test* for multiple comparisons. Differences from the controls were considered significant as *p < 0.05, **p < 0.001, ***p < 0.0001. GraphPad Prism 5.03 version software was used for all statistical assessments.

## Results

### Mutagenicity/genotoxicity

Before the assessment of the protective effects of *F*. *angustifolia* Vahl. extracts, Ames and *umu* tests were also used to study, respectively, the eventual mutagenic and genotoxic effects of these extracts. At 1000 μg/mL, they did not reveal any mutagenic effect, as reported in S1 and S2 Figs. In fact, in *S*. *typhimurium* TA 98 and TA 100 strains, there were observed neither direct/indirect frame-shift mutations nor direct/indirect missense mutations (M R < 2) were observed. Similarly, no direct/indirect genotoxic effects were observed (S1 Table) indicating no induction of the SOS-repair system in the *S*. *typhimurium* TA1535/pSK1002 strain (IR < 1.5).

### Antimutagenicity

Results of antimutagenic activity with and without metabolic activation are presented respectively in Tables 1 and 2, as the mean of revertants/plates ± SD of three independent experiments (each one based on three replicates). Inhibition rate percentage was calculated [29]. Basically, significant moderate and strong antimutagenic effects exerted by *F*.*angustifolia* Vahl. leaves and stem bark extracts were observed in *S*. *typhimurium* strains. Three concentrations (25, 50 and 100 μg/mL) of extracts were co-incubated for 72h with standard direct mutagens. Altogether, inhibition rate percentage values, calculated for TA100, were higher than those of TA98 (Table 1).

**Table 1:** Direct antimutagenic activities of *F*. *angustifolia* Vahl. Antimutagenic activities of *F*. *angustifolia* Vahl. leaves and stem bark extracts on TA98 and TA100 strains after 72h co-incubation with direct mutagens, respectively 2-NF (2.5 and 5 μg/mL) and NaN_3_ (5 and 10 μg/mL), in the absence of metabolic activation system S9.

Treatment [μg/mL]		TA 98 revertants/plate (mean±SD)		Inhibition rate (%)		TA 100 revertants/plate (mean±SD)		Inhibition rate (%)	
		2-NF 2.5μg/mL	2-NF 5μg/mL	2-NF 2.5μg/mL	2–5μg/mL	NaN_3_ 5μg/	mLNaN_3_ 10μg/mL	NaN_3_ 5μg/mL	NaN_3_ 10μg/mL
**FL1**	25	77 ± 4 ***	183 ± 79*	52^c^	46^c^	237 ± 25 ***	283 ± 50 ***	49^c^	56^c^
	50	88 ± 16 ***	163 ± 59 *	46^c^	52^c^	282 ± 20 ***	345 ± 7 ***	39^b^	47^c^
	100	99 ± 10 ***	233 ± 3	39^b^	32^b^	250 ± 3 ***	354 ± 8 ***	46^c^	45^c^
**FL2**	25	94 ± 17 ***	333 ± 22	42^c^	2^a^	252 ± 19 ***	381 ± 14 ***	45^c^	41^c^
	50	96 ± 8 ***	330 ± 44	41^c^	3^a^	222 ± 6 ***	379 ± 13 ***	52^c^	41^c^
	100	98 ± 16***	336 ± 11	40^b^	1^a^	213 ± 21 ***	352 ± 18 ***	54^c^	46^c^
**FL3**	25	105 ± 3**	175 ± 72 *	35^b^	49^c^	261 ± 27 ***	306 ± 6 ***	43^c^	53^c^
	50	95 ± 9 ***	324 ± 62	41^c^	5^a^	242 ± 59 ***	302 ± 14 ***	48^c^	53^c^
	100	98 ± 10 ***	240 ± 14	40^b^	30^b^	267 ± 4 ***	348 ± 40 ***	42^c^	46^c^
**FL4**	25	77 ± 4 ***	210 ± 13	52^c^	38^b^	290 ± 18 ***	339 ± 44 ***	37^b^	48^c^
	50	86 ± 16 ***	279 ± 13	47^c^	18^a^	349 ± 92	388 ± 25 ***	24^a^	40^b^
	100	88 ± 4 ***	273 ± 16	46^c^	20^a^	252 ± 17 ***	382 ± 48 ***	45^c^	41^c^
**FL5**	25	96 ± 7 ***	330 ± 17	41^c^	3^a^	277 ± 73 ***	301 ± 40 ***	40^b^	53^c^
	50	98 ± 17 ***	182 ± 73 *	40^b^	47^c^	374 ± 9	304 ± 6 ***	19^a^	53^c^
	100	111 ± 9 **	256 ± 40	31^b^	25^b^	327 ± 16 **	308 ± 7 ***	29^b^	52^c^
**FB1**	25	81 ± 4 ***	193 ± 80	50^c^	43^c^	225 ± 23 ***	368 ± 5 ***	51^c^	43^c^
	50	102 ± 17 **	332 ± 14	37^b^	3^a^	258 ± 8***	367 ± 4 ***	44^c^	43^c^
	100	89 ± 11 ***	240 ± 28	45^c^	30^b^	296 ± 19***	354 ± 3 ***	36^b^	45^c^
**FB2**	25	71 ± 13 ***	306 ± 8	56^c^	10^a^	248 ± 11 ***	365 ± 17 ***	46^c^	44^c^
	50	108 ± 17 *	332 ± 6	33^b^	3^a^	244 ± 34 ***	314 ± 42 ***	47^c^	51^c^
	100	83 ± 16 ***	334 ± 0	49^c^	2^a^	264 ± 23 ***	300 ± 96 ***	43^c^	54^c^
**FB3**	25	80 ± 4 ***	218 ± 10	51^c^	36^b^	274 ± 41 ***	314 ± 4 ***	41^c^	51^c^
	50	106 ± 6 *	173 ± 71 *	35^b^	33^b^	250 ± 5 ***	372 ± 17 ***	46^c^	43^c^
	100	75 ± 4 ***	230 ± 19	54^c^	37^b^	262 ± 2 ***	350 ± 14 ***	43^c^	46^c^
**FB4**	25	109 ± 29 *	323 ± 10	33^b^	5^a^	258 ± 47 ***	358 ± 7 ***	44^c^	45^c^
	50	105 ± 9 **	191 ± 84	35^b^	44^c^	298 ± 60 ***	348 ± 17 ***	35^b^	46^c^
	100	76 ± 8 ***	271 ± 58	53^c^	21^a^	366 ± 16	355 ± 33 ***	21^a^	45^c^
**FB5**	25	103 ± 27 **	279 ± 66	36^b^	18^a^	292 ± 6 ***	351 ± 51 ***	37^b^	46^c^
	50	110 ± 6 *	211 ± 86	32^b^	38^b^	276 ± 11 ***	326 ± 20 ***	40^b^	50^c^
	100	113 ± 6 *	154 ± 54**	30^b^	55^c^	306 ± 89 ***	334 ± 20 ***	34^b^	48^c^
**M**		162±44	341±79	-	-	461±43	647±16	-	-
**NC**		60 ± 7		-	-	220±25		-	-

Results are expressed as mean of revertants/plates ± SD (three independent experiments). Inhibition rate percentage was calculated as follows: 100- [(T/M) x 100] where T is the mean number of revertant colonies in the plate containing both mutagen and tested extract, and M is the mean number of revertant colonies in the plate containing only the mutagen [29]. Significant difference for *p <0.05, **p< 0.01, *** p < 0.001 (*Dunnett’s test*) was calculated comparing extracts co-treated with standard mutagens to single standard mutagens.

**NC** negative control; **M** mutagen; **2-NF** 2- nitrofluorene; **NaN**_**3**_ Sodium Azide **FL** *F*.*angustifolia* Vahl. leaves; **FB** *F*.*angustifolia* Vahl. stem bark

**1** Ethanolic; **2** Organic/Ethyl Acetat; **3** Aqueous/ Ethyl Acetat; **4** Organic/ Chloroform; **5** Aqueous/ Chloroform

^a^No antimutagenic effect (< 25% inhibition)

^b^Moderate effect (25%– 40% inhibition)

^c^Strong antimutagenic effect (> 40% inhibition) [29].

**Table 2:** Indirect antimutagenic effects of *F*. *angustifolia* Vahl. Antimutagenic effects of *F*. *angustifolia* Vahl. leaves and stem bark extracts on strains TA98 and TA100 after 72h co-incubation with indirect mutagens, respectively 3-MC (25 and 50 μg/mL) and CP (50 and 100 μg/mL), in presence of metabolic activation system S9.

	TA 98				TA 100			
Treatments 25μg/mL	Mean revertants/plate±SD		Inhibition rate (% mean±SD)		mean revertants/plate±SD		Inhibition rate (% mean ±SD) ^b^	
	3-MC 25μg/mL	3-MC 50μg/mL	3-MC 25μg/mL	3-MC 50μg/mL	CP 50μg/mL	CP 100μg/mL	CP 50μg/mL	CP 100μg/mL
**FL1**	79 ± 5 ***	158 ± 1 ***	40 ± 4^b^	32± 3^b^	244 ± 13 ***	484± 13***	43 ± 5^c^	29 ± 3^b^
**FL2**	87 ± 2 ***	173 ± 6 ***	34 ± 3^b^	26± 4^b^	226± 5***	439± 23***	47± 4^c^	35± 4^b^
**FL3**	89 ± 2 ***	190 ± 2 ***	32± 3^b^	18 ± 4^a^	251± 8***	458± 2***	41 ± 5^c^	32± 2^b^
**FL4**	76± 2 ***	155 ± 4 ***	42± 3^c^	33 ± 3^b^	300± 16 ***	467 ± 7***	29 ± 6^c^	31± 2^b^
**FL5**	76± 0 ***	151± 6 ***	42 ± 2^c^	35± 4^b^	252± 7 ***	455± 4 ***	41 ± 5^c^	33 ± 2^b^
**FB1**	84 ± 2 ***	155± 2 ***	36 ± 3^b^	33± 3^b^	263± 13 ***	460 ± 13***	38 ± 5^c^	32 ± 3^b^
**FB2**	75± 1 ***	152± 15 ***	43 ± 2^c^	35 ± 7^b^	240 ± 6***	506± 30 ***	44 ± 4^c^	25 ± 5^b^
**FB3**	84± 1***	171 ± 1 ***	36± 3^b^	27 ± 3^b^	231± 6***	490 ± 18 ***	46 ± 4^c^	28 ± 3^b^
**FB4**	88± 2 ***	168± 2 ***	33 ± 3^b^	28 ± 3^b^	235 ± 6***	443± 18 ***	45 ± 4^c^	35 ± 3^b^
**FB5**	80 ± 5 ***	160 ± 2***	39 ± 4^b^	31 ± 3^b^	229± 12***	436 ± 21 ***	46 ± 5^c^	36 ± 4^c^
**M**	131 ± 5	233± 10	-	-	425± 31	677 ± 18	-	-
**NC**	60± 7	-	-	-	220± 25	-	-	-

Results are expressed as mean of revertants/plates ± SD (three independent experiments). Inhibition rate percentage was calculated as follows: 100- [(T/M) x 100] where T is the mean number of revertant colonies in the plate containing both mutagen and tested extract, and M is the mean number of revertant colonies in the plate containing only the mutagen [25]. Significant difference for ***p<0.001 (*Dunnett’s test*) was calculated comparing extracts co-treated with standard mutagens to single standard mutagens.

**NC** negative control; **M** mutagen**; 3MC** 3-Metilcolanthrene; **CP** Cyclophosphamide; **FL** *F*.*angustifolia* Vahl. leaves; **FB** *F*.*angustifolia* Vahl. stem bark

**1** Ethanolic; **2** Organic/Ethyl Acetat; **3** Aqueous/ Ethyl Acetat; **4** Organic/ Chloroform; **5** Aqueous/ Chloroform

^a^No antimutagenic effect (< 25% inhibition)

^b^Moderate effect (25%– 40% inhibition)

^c^Strong antimutagenic effect (> 40% inhibition) [29].

Thus, extracts were mainly able to inhibit the direct mis-sense mutations caused by both concentrations of NaN_3_ (5 and 10 μg/mL) in TA 100, but were less active in inhibiting direct frame-shift mutations towards the higher concentration of 2-NIT (5 μg/mL) in TA 98. Specifically, FL2, FL4, FB1, FB2, FB4 did not induce significant inhibition of the frame-shift mutations induced by 2-NIT (5 μg/mL). Indirect antimutagenicity experiments, were performed only at 25 μg/mL of extracts, which was the lowest effective concentration obtained in direct antimutagenicity experiments. Significant moderate and strong antimutagenic effects (***p < 0.0001) were observed for all extracts in presence of metabolic activation (Table 2). Hence, protective effect towards indirect frame- shift and missense mutations was exhibited, respectively in both TA98 and TA100, at both concentrations of standard indirect mutagens (3-MC: 25 and 50 μg/mL; CP: 50 and 100 μg/mL).

### Antigenotoxicity

Antigenotoxicity results, due to the properties of the extracts in inhibiting the SOS response in *S*. *typhimurium* TA1535/pSK1002, were expressed as mean of induction ratio (IR) ± SD (n = 3), and are reported in Table 3. Significant differences (**p< 0.001, *** p < 0.0001) were observed between IR values obtained from extracts co-treated with direct standard genotoxin (4-NQO, 0.05 μg/mL) and IR values obtained from the single 4-NQO. Antigenotoxicity (% mean ± SD), calculated [31], revealed a moderate or strong effect induced by all extracts towards the direct genotoxin in absence of metabolic activation, with the highest inhibition by FL5 and FB5 (antigenotoxicity by 70%). On the contrary, in presence of metabolic activation, FL3, FB4 and FB5 showed a neutral effect (Antigenotoxiciy less than 40%), while FL1, FL2, FL4 and FB1 showed significant (**p< 0.001, ***p < 0.0001) moderate to strong protective effect against the indirect genotoxin (2AA, 0.2 μg/mL).

**Table 3:** Antigenotoxicity of *F*. *angustifolia* Vahl. Antigenotoxicity of *F*. *angustifolia* Vahl. leaves and stem bark extracts (25, 50, 100 μg/mL) after 2h co-incubation with standard genotoxins: 4-NQO 0.05μg/mL and 2-AA 0.20μg/mL, respectively for the treatment in absence and in presence of metabolic activation S9.

**Treatment [μg/mL]**		**IR ± SD**			
		**-S9**		**+S9**	
**NC**	0	1.00 ± 0.00		1.00 ± 0.00	
**4-NQO**	0.05	3.13 ± 0.34		-	
**2-AA**	0.20	-		4.56 ± 0.66	
		**+ 4-NQO**		**+ 2-AA**	
		**IR ± SD**	**Antigenotoxicity (% mean ± SD)**	**IR ± SD**	**Antigenotoxicity (% mean ± SD)**
**FL1**	25	1.60±0.24***	48.90 ± 0.29^b^	2.09± 0.36**	54.00 ± 1.72^b^
	50	1.56±0.17***	50.10 ± 2.25^b^	2.46± 0.42	45.73 ± 1.93^b^
	100	1.82±0.30**	42.05 ± 0.68^b^	2.35± 0.33*	48.20 ± 3.34^b^
**FL2**	25	1.13±0.04***	63.50 ± 4.55^b^	1.60± 0.48***	65.29 ± 3.40^b^
	50	1.10±0.10***	64.63 ± 2.00^b^	1.77± 0.35**	61.20±0.34^b^
	100	1.12±0.22***	64.22 ± 1.56^b^	1.57± 0.35***	65.56 ± 0.52^b^
**FL3**	25	1.75±0.47**	44.55 ± 6.50^b^	2.92± 0.40	35.55 ± 4.67^a^
	50	1.69±0.21**	46.05 ± 1.55^b^	3.25± 0.74	28.95 ± 1.59^a^
	100	1.74±0.32**	44.47 ± 1.69^b^	2.60± 0.13	41.93 ± 9.10^b^
**FL4**	25	1.24±0.23***	60.41 ± 1.25^b^	1.87± 0.68**	59.60 ± 6.65^b^
	50	1.15±0.03***	62.66 ± 6.78^b^	2.08± 0.71**	55.10 ± 6.23^b^
	100	1.23±0.13***	60.64 ± 2.39^b^	1.64± 0.38***	64.16 ± 0.95^b^
**FL5**	25	0.92±0.03***	70.31 ± 5.56^c^	1.92± 0.23**	57.44 ± 3.73^b^
	50	1.13±0.02***	63.50 ± 4.94^b^	2.28 ± 0.10*	49.17 ± 8.23^b^
	100	1.03±0.04***	66.84 ± 3.80^b^	2.47± 0.38	45.52 ± 3.00^b^
**FB1**	25	1.37±0.50***	57.02 ± 9.30^b^	2.26± 0.82*	51.16 ± 7.96^b^
	50	1.38±0.33***	56.21 ± 3.87^b^	2.50± 0.88	46.02 ± 8.08^b^
	100	1.41±0.32***	55.09±3.27^b^	1.89± 0.40**	58.49 ± 0.16^b^
**FB2**	25	1.19±0.26***	62.13 ± 2.45^b^	2.47± 0.30	45.31 ± 4.72^b^
	50	1.23±0.18***	60.69 ± 021^b^	2.27± 0.06*	49.35 ± 9.25^b^
	100	1.43±0.24***	54.35±0.67^b^	2.66± 0.16	40.72 ± 8.65^b^
**FB3**	25	1.20±0.23***	61.93 ± 1.48^b^	2.40± 0.58*	47.52 ± 1.86^b^
	50	1.28±0.32***	59.49 ± 3.96^b^	2.60± 0.71	43.36 ± 3.84^b^
	100	1.47±0.48***	53.80±8.08^b^	2.45± 0.71*	46.71 ± 4.53^b^
**FB4**	25	1.26±0.36***	60.08 ± 5.24^b^	2.93± 0.93	36.48 ± 7.18^a^
	50	1.38±0.16***	55.64 ± 1.71^b^	2.87± 0.28	36.37 ± 6.92^a^
	100	1.45±0.12***	53.49±3.39^b^	2.43± 0.10*	45.70 ± 9.00^b^
**FB5**	25	1.12±0.22***	64.26 ± 1.41^b^	2.66± 0.25	40.87 ± 6.64^b^
	50	1.17±0.16***	62.45 ± 0.84^b^	2.91± 0.17	35.11 ± 9.65^a^
	100	1.00±0.11***	67.94±1.37^b^	2.63± 0.24	41.50 ± 6.94^b^

Results are expressed as mean of induction ratio (IR) ± SD (n = 3) with significant difference for *p <0.05, **p< 0.01, *** p < 0.001 (*Dunnett’s test*) calculated comparing IR values obtained from extracts co-treated with standard genotoxins to IR values obtained from single standard genotoxins. Antigenotoxicity (% mean ± SD) was calculated as follows: [1-(βgalactosidase unit _GENOTOXIN+SAMPLE_ / βgalactosidase unit _GENOTOXIN_)] *100%, [31].

**NC** negative control; **4-NQO** 4-nitroquinoline; **2AA** 2-amino-anthracene

**FL** *F*.*angustifolia* Vahl. leaves; **FB** *F*.*angustifolia* Vahl. stem bark

**1** Ethanolic; **2** Organic/Ethyl Acetat; **3** Aqueous/ Ethyl Acetat; **4** Organic/ Chloroform; **5** Aqueous/ Chloroform

^a^Neutral effect (< 40% Antigenotoxicity)

^b^Moderate effect (40%– 70% Antigenotoxicity)

^c^Strong effect (>70% Antigenotoxicity) [32].

### Antiproliferative activity

Results regarding the potential antiproliferative activity exerted after 72h (MTT assay) by *F*. *angustifolia* Vahl. extracts on Hep-G2 and MCF-7 cell lines were expressed as IC_50_ values and are reported in Table 4. Almost completely overlapped 95% confidence intervals of IC_50_ values were found in all extracts. In addition, the lowest observable adverse effect concentrations (LOAECs, *p* < 0.05) were observed with FL5 (125 μg/mL), FB3 and FB5 (250 μg/mL) on Hep-G2, while FB4 (100 μg/mL), FB5 (125 μg/mL), FL1 and FL5 (250 μg/mL) were obtained on MCF-7. Furthermore, IC_50_ values of the antiproliferative activity exerted by the extracts on normal-like cells (TelCOFS02MA) were used to calculate SI [34]. Selective toxicity towards cancer cells was shown by FL1 (SI: 2.04, Hep-G2; 2.93, MCF-7), FL5 (SI: 2.40, Hep-G2; 2.21, MCF-7) and FB5 (SI: 2.52, Hep-G2; 2.90, MCF-7).

**Table 4:** MTT assay. IC_50_ values (μg/mL), with 95% confidence range (in brackets) obtained by MTT assay after 72h treatment of Hep-G2 and MCF7 cell lines with leaves and stem bark extracts of *F*.*angustifolia* Vahl.

Treatment	Hep-G2		MCF-7	
	IC_50_ (confidence range)	LOAEC	IC_50_ (confidence range)	LOAEC
**FL1**	713 (488–1041)	500	496 (281–871)	250
**FL2**	527 (326–853)	500	1047 (884–1240)	750
**FL3**	1644 (1453–1862)	1000	1053 (824–1344)	1000
**FL4**	680 (372–1241)	500	970 (732–1287)	500
**FL5**	441 (242–801)	125	479 (352–651)	250
**FB1**	530 (303–929)	500	682 (469–942)	500
**FB2**	626 (498–787)	500	886 (645–1218)	750
**FB3**	431 (286–649)	250	882 (627–1240)	750
**FB4**	777 (664–908)	500	634 (631–932)	100
**FB5**	504 (411–617)	250	437 (330–578)	125

LOAEC: Lowest Observable Adverse Effect Concentration (μg/mL), (*Dunnett’s test*, *p* < 0.05)

**FL** *F*.*angustifolia* Vahl. leaves; **FB** *F*.*angustifolia* Vahl. stem bark

**1** Ethanolic; **2** Organic/Ethyl Acetat; **3** Aqueous/ Ethyl Acetat; **4** Organic/ Chloroform; **5** Aqueous/ Chloroform

### Phytochemical investigation

#### Solvent extraction yield and quantification of total phenolics and flavonoids

Extraction yield from raw plant material, total phenolics and flavonoids are reported in Table 5. Ethanolic extracts FL1 and FB1 exhibited a high percentage, 8.5 and 5.9%, respectively. While, the aqueous/chloroform extract FL5 and FB5 showed the lowest percentages (0.7; 0.5%) among all other extracts.

**Table 5:** Extraction yield and quantitative phytochemicals screening. Extraction yield and quantitative phytochemicals screening of extracts from *F*. *angustifolia* Vahl.

*F*. *angustifolia* Vahl.	Extraction Yield (g dry extract/100g of powder)	Extraction content	
		Polyphenols (μg Cat. Eq. /mg dry extract)	Flavonoids (μg Rut. Eq. /mg dry extract)
**FL1**	8.53	98.63 ± 6.25	16.22 ± 5.02
**FL2**	3.88	105.95 ± 1.45^ns^	16.67 ± 1.20^ns^
**FL3**	5.56	44.94 ± 4.38***	15.90 ± 2.62^ns^
**FL4**	5.2	120.65 ± 2.83***	16.67 ± 1.20^ns^
**FL5**	0.74	99.52 ± 1.65^ns^	15.63± 2.91^ns^
**FB1**	5.85	92.86 ± 2.91	11.68 ± 0.49
**FB2**	1.32	76.13 ± 0.94^###^	15.93 ± 5.09^ns^
**FB3**	4.25	75.30 ± 1.23^###^	10.61 ± 0.96^ns^
**FB4**	3.18	32.56 ± 0.94^###^	17.56 ± 3.55^ns^
**FB5**	0.48	207.08 ± 4.83^###^	14.25±3.02^ns^

Data are presented as means ± SD (n = 3). μg Cat. Eq.: microgramme catechin equivalent, μg Rut. Eq.: microgramme rutin equivalent.

^ns^ Not significant

***p<0.001 compared to FL1

^###^p<0.001 compared to FSB1.*,# Significance (p<0.001) by One way ANOVA test, with Dunnett’s post test of GraphPad Prism Software. **FL** *F*.*angustifolia* Vahl. leaves; **FB** *F*.*angustifolia* Vahl. stem bark; **1** Ethanolic; **2** Organic/Ethyl Acetat; **3** Aqueous/ Ethyl Acetat; **4** Organic/Chloroform; **5** Aqueous/ Chloroform

All extracts exhibited the presence of various quantities of total phenolics and flavonoids contents. Leaves extracts contained approximately the same amount of total phenols as the corresponding stem bark extract, while the most prominent being that of the aqueous chloroform fractions (FB5) with 207 μg catechin equivalent/ mg dry extract (Cat. Eq. mg^-1^extract). On the other hand, no differences were noticed for flavonoids contents. In fact, the amount ranged from 15.63 to 16.67 μg Rutin Eq mg^-1^ extract for leaves, and from 10.61 to 17.56 μg Rutin Eq mg^-1^ extract for stem bark.

**Fig 1:**
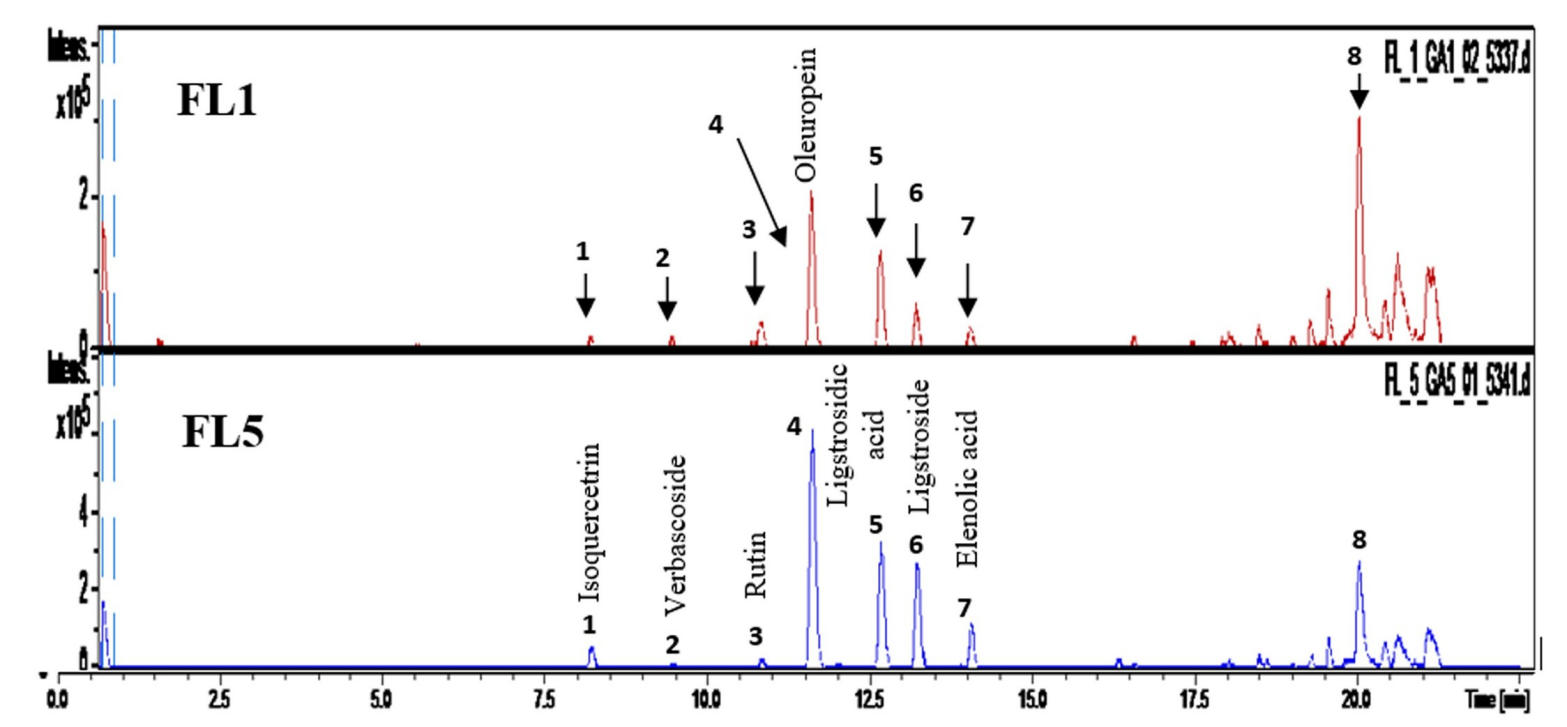
Leaves HPLC chromatogram. HPLC chromatogram (254 nm) of the composition of the aqueous chloroform (FL5) extract and its native crude extract (FL1) of *F*. *angustifolia* Vahl leaves. FL1: Ethanolic extract; FL5: aqueous/ chloroform extract.

*UHPLC/MS investigation*. The UHPLC/MS analysis was carried out in negative mode, phenolic compounds were identified by examination of retention times, UV -visible and mass spectra, corresponding to the different metabolites eluted from leaves and stem bark extracts of *F*. *angustifolia* Vahl. the UHPLC chromatograms of the aqueous extract of chloroform (FL5) and its native ethanolic extract (FL1) are presented in Fig 1, the molecular weights and retention times collected from the analysis together with the identifications of the detected metabolites are shown in Table 6.

**Table 6:** Retention times (t_*r*_) of the ions (*m/z*) and the molecular weights. Table of the retention times (t_*r*_) of the ions (*m/z*) and the molecular weights corresponding to the peaks revealed in the HPLC/MS spectra of *F*. *angustifolia* Vahl. leaves extracts.

Extract	N°	t_R_ (min)	*m*/z Experimental	Max MW	Intensity	Molecular formula	Identified metabolite
**FL1**	1	1.0	181.10	182.11	20816	C_9_H_10_O_4_	Syringaldehyd
	2	7.9	463.19	464.20	28659	C_21_H_20_O_12_	Isoquercitrin
	3	10.5	609.20	610.21	52629	C_27_H_30_O_16_	Rutin
	**4**	**11.4**	**539.23**	**540.23**	**236032**	**C**_**25**_**H**_**32**_**O**_**13**_	**Oleuropein**
	**5**	**12.5**	**569.24**	**570.25**	**266002**	---	**Ligstrosidic acid**
	6	13.1	523.24	524.24	98953	C_25_H_32_O_12_	Ligstroside
	7	14.0	601.27	602.27	43358	---	Derivative of Elenolic acid
	8	16.5	909.38	910.39	28220	---	Isomer of GL5
	9	20.3	698.97	699.98	80336	---	Gallic acid dihexoside sinapoyl
	**10**	**20.4**	**532.97**	**533.98**	**117390**	**C**_**24**_**H**_**22**_**O**_**14**_	**Kaempferol-3-*O*-malonyl glucoside**
	11	21.0	255.26	256.27	27040	C_15_H_12_O_4_	Liquirtigenin
**FL5**	1	7.9	463.19	464.19	30623	C_21_H_20_O_12_	Isoquercitrin
	2	9.2	623.25	624.26	12982	C_29_H_36_O_15_	Verbascoside /Isoverbascoside
	3	10.5	609.20	610.21	16134	C_27_H_30_O_16_	Rutin
	**4**	**11.4**	**539.22**	**540.23**	**392912**	**C**_**25**_**H**_**32**_**O**_**13**_	**Oleuropein**
	**5**	**12.5**	**569.24**	**570.24**	**276806**	**C**_**26**_**H**_**34**_**O**_**14**_	**Ligstrosidic acid**
	**6**	**13.1**	**523.23**	**524.24**	**208500**	**C**_**25**_**H**_**32**_**O**_**12**_	**Ligstroside**
	7	14.0	601.26	602.27	69027	---	Derivative of Elenolic acid

**FL**: *F*. *angustifolia* Vahl. leaves; **1**: Ethanolic extract; **5**: Aqueous/chloroform extract

**In Bold**: identified metabolite with high intensity

The shared metabolites between these two extracts, with more intensities in the aqueous phase (in bold, Table 6) are in the first place, oleuropein (4), a major compound, common to Oleaceae family (especially *Fraxinus* species) derived from the biosynthetic pathway of a secoiridoid [37], its main metabolites are tyrosol and hydroxytyrosol [38]. Two other metabolites belonging to this same chemical group, ligtroside (6) with an intensity twice as high in the aqueous extract as the ethanolic extract and the compound (5) supposed to be ligstrosidic acid, according to the bibliographic data corresponding to the results of the UHPLC / MS analysis of the fraction where this metabolite is the major one. It is also remarkable that these two extracts share two flavonols glycosides, isoquercitrin (1) and rutin (3), they are glycoside derivatives of quercetin, which were previously reported as the constituents of genus *Fraxinus* has been reported [39]. There is also a compound whose intensity is not very different, elenolic acid (7), which would come from the hydrolysis of oleuropein which releases hydroxytyrosol and elenolic acid. Tyrosol, on the other hand, comes from the degradation of ligstroside [40]. Finally, we note the presence of a low intensity peak of a phenylethanoid, verbascoside (2), previously reported in several *Fraxinus* species.

**Fig 2:**
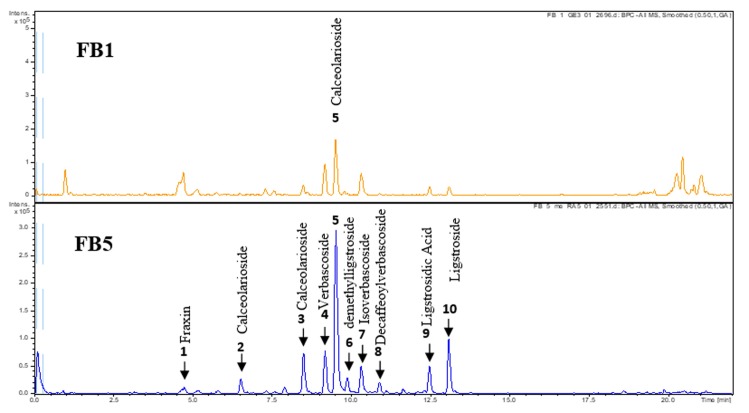
Bark HPLC chromatogram. HPLC chromatogram (254 nm) of the composition of the aqueous chloroform (FSB5) extract and its native crude extract (FSB1) of *F*. *angustifolia* Vahl stem bark. FB1 ethanolic extract; FB5 aqueous/ chloroform extract.

HPLC chromatograms and identified metabolites in the aqueous extracts of chloroform (FB5) and its native ethanolic extract (FB1) are Illustrated in Fig 2 and Table 7.

**Table 7:** Retention times (t_*r*_) of the ions (*m/z*) and the molecular weights. Table of the retention times (t_*r*_) of the ions (*m/z*) and the molecular weights corresponding to the peaks revealed in the HPLC/MS spectra of *F*. *angustifolia* Vahl. stem bark extracts.

Extract	N°	t_R_ (min)	*m*/z Experimental	Max MW	Intensity	Molecular formula	Identified metabolite
**FB 1**	1	1.0	341.15	342.15	81408	C_15_H_18_O_9_	Caffeic acid glucoside
	2	4.7	369.13	370.13	68630	C_16_H_18_O_10_	Fraxin
	3	5.1	429.15	430.15	20964	---	---
	4	7.3	535.23	536.24	21362	C_26_H_32_O_12_	8-Hydroxypinoresinol-4/8-glucoside.
	5	8.5	477.18	478.19	30486	C_23_H_25_O_11_	Calceolarioside A
	6	9.2	623.25	624.26	95389	C_29_H_36_O_15_	Verbascoside
	**7**	**9.5**	**477.19**	**478.19**	**174206**	**C**_**23**_**H**_**25**_**O**_**11**_	**CalceolariosideB**
	8	10.3	623.25	624.26	67733	C_29_H_36_O_15_	Isoverbascoside
	9	12.5	569.24	570.25	26693	C_26_H_34_O_14_	Ligstrosidic acid
	10	13.1	523.23	524.24	28317	C_25_H_32_O_12_	Ligstroside
	11	19.6	269.22	270.23	21594	C_8_H_13_O_10_	Apigenin
	12	20.3	698.97	699.98	68940	---	Gallic acid dihexosidesinapoyl
	**13**	**20.5**	**532.98**	**533.98**	**120273**	**C**_**24**_**H**_**22**_**O**_**14**_	**Kaempferol-3-*O*-malonyl glucoside**
	14	21.0	255.26	256.27	62872	C_15_H_12_O_4_	Liquirtigenin
**FB 5**	1	4.7	369.12	370.13	10714	C_16_H_18_O_10_	Fraxin
	2	6.5	477.18	478.19	27245	C_23_H_25_O_11_	Calceolarioside
	3	7.9	477.18	478.19	12107	C_23_H_25_O_11_	Calceolarioside
	4	9.2	623.25	624.26	81456	C_29_H_36_O_15_	Verbascoside
	**5**	**9.5**	**477.18**	**478.19**	**299210**	**C**_**23**_**H**_**25**_**O**_**11**_	**Calceolarioside B**
	6	9.9	509.21	510.22	27567	C_24_H_30_O_12_	demethyligstroside
	7	10.3	623.25	624.26	50994	C_29_H_36_O_15_	Isoverbascoside
	8	10.9	461.19	462.19	19841	C_20_H_29_O_12_	Decaffeoylverbascoside
	9	12.5	569.24	570.24	51995	C_26_H_34_O_14_	Ligstrosidic acid
	**10**	**13.1**	**523.23**	**524.23**	**102076**	**C**_**25**_**H**_**32**_**O**_**12**_	**Ligstroside**

**FB**: *F*. *angustifolia* Vahl. stem bark; **1**: Ethanolic extract; **5**: Aqueous/chloroform extract

**In Bold**: identified metabolite with high intensity

It is noted in this comparative figure that FB5 concentrated and selected mainly phenylethanoids and secoiridoids compared to the more diverse composition in metabolites with lower incidences in the native extract (FB1). The two major compounds of this extract are calceolarioside B and ligstroside. Their isomers and derivatives, namely at least two isomers of calceolarioside B, the verbascoside and its isoverbascoside isomer, and its decaffeoyl derivative. Similarly, lower levels were found for ligstroside, demethyl-ligstroside and ligstrosidic acid. This extract also selected a small amount of a coumarin common in the genus *Fraxinus*, fraxin.

## Discussion

Too often, treatments based on medicinal plants can cause serious health risks [41]. Thus, it was consistent to assess the mutagenic **and** genotoxic **activities** of *F*. *angustifolia* Vahl. extracts in order to exclude their eventual negative impact on genetic material.

In this initial report we demonstrated that the tested *F*. *angustifolia* Vahl. extracts did not produce DNA lesions. The absence of genotoxicity/mutagenicity is not typical of all natural products in use, since several medicinal plants, assayed with the *umu* and/or the Ames test, in the presence or absence of the S9 metabolic activation, proved to be positive for genotoxicity/mutagenicity [32, 42–44]. Polyphenols are a diverse class of compounds; many are favorable in preventing disease and protecting the stability of the genome. However not all polyphenols and not all their actions are necessarily advantageous. Some of them have mutagenic and/or pro-oxidant effects. It was proven that several polyphenols, including quercetin, can bind to DNA and this direct interaction may be an important mechanism of bacterial mutagenicity, even those polyphenols that are negative in bacterial systems may be clastogenic in mammalian cells [45].

Furthermore, conflicting results about the mutagenic potential of verbascoside, a phenylethanoid compound, identified in *F*. *angustifolia* Vahl. when tested on TA 98 and TA 100, did not induce frameshift and base-pair substitution mutations in the presence or absence of metabolic system [46], but induced DNA damages on human lymphocytes, with an involvement of PARP-1 and p53 expression through its caffeic acid moiety [47].

Recently, *in vivo* subsequent tests on *Drosophila melanogaster* and rabbit reported that verbascoside do not give rise to any mutagenic activity at any of the tested concentrations [48–50]. Besides poor *in vivo* bioavailability of verbascoside was reported and could be also related to this behavior [51].

Substances with antigenotoxic **and/or** antimutagenic properties may be useful against the damage caused by environmental mutagens. The antigenotoxic study of *F*. *angustifolia* Vahl. extracts was first evaluated by the *umu* test towards the direct mutagen 4-NQO, revealing to have a strong antigenotoxic effect (FL5 and FB5 extracts, inhibition by 70%) besides a moderate effect observed with the indirect mutagen 2-AA. This indicates that these extracts may not be involved in 2-AA genotoxic action but in the 4-NQO mutagenic mechanism.

4-NQO induces intracellular oxidative stress; it can undergo redox cycling and generate reactive oxygen species such as superoxide radical and hydrogen peroxide [52, 53]. Furthermore, *F*. *angustifolia* Vahl. has revealed a potent antioxidative capacity [19, 26]; Hence, the possible mechanism of the observed antigenotoxic potency could involve the bioactive phenolic antioxidant compounds present in the *F*. *angustifolia* Vahl. extracts which might interact with the reactive intermediates of 4-NQO.

The second known mechanism of the carcinogenic action of 4-NQO is the formation of stable mono-adducts on purines [54, 55]. This damage could be repaired by the nucleotide excision repair pathway [54]. Moreover, the strain TA1553 /pSK 1002 is *uvrb* deficient [56]. Taken altogether, these results indicate that, in addition to antioxidant activity *F*.*angustifolia* Vahl., leaves and stem bark extracts may exert their antimutagenic properties also by modulating the DNA repair processes [57].

*Umu* test showed lower detection sensitivities to the weaker Ames positive chemicals of relatively strong Ames positive [58]. Moreover, Oda in 2016 [59] concluded that *umu* and Ames test are complementary and would be used together in order to broaden the detection capacity. In fact, *umu* test detects damage to DNA by evaluating the expression level of the *umu* gene product, which is one of the DNA repairing enzymes induced through the SOS response while Ames assay detects the mutation of gene for histidine synthesis by growing a mutant colony that is a phenotype of such mutation [58].

In the present study, a variation of Ames test was used to assess the antimutagenic activity of *F*.*angustifolia* Vahl. extracts against other mutagens (2-NF, NaN_3_, 3-MC and CP) with different mutagenic mechanism than that used for *umu* test. In addition, extracts of *F*. *angustifolia* Vahl. were subjected to two concentrations of the standard mutagens for better understanding of the dose effect relation.

*F*. *angustifolia* Vahl. leaves and stem bark extracts exhibited a protective effect against low concentrations of mutagens, on both strains TA98 and TA100 independently tested with or without metabolic activation. Regarding high concentrations of standard mutagen, the extracts were effective on TA100 and less active on TA98, when tested in the absence of metabolic activation. The extracts acted by interrupting several mutagenesis processes, indicating that they may directly protect the DNA from damages, possibly by quick elimination of mutagens from the bacteria before their interaction with the DNA. They may also facilitate or stimulate the bacterial transmembrane export system to eliminate the mutagens, or interfere with the uptake of mutagens into bacteria [57, 60]. However, the inhibition of mutagenesis is often complex, involving multiple mechanisms [61].

Otherwise, cytotoxicity and selectivity of *F*. *angustifolia* Vahl. were examined on normal-like cells (human fibroblasts, TelCOFS02MA), human hepatocarcinoma cells (HepG2) and human adenocarcinoma cells (MCF-7). Overall, it is interesting to observe a correlation between phytochemicals profile identified by HPLC/MS and the selectivity degree of the extracts. FL1 and FL5 were selective towards the two cancer cell lines, and this may be due to the high content of oleuropein and ligstrosidic acid detected in the two extracts; oleuropein is a characteristic phenol largely distributed in Oleaceae family, and involved in a number of exhaustively documented biological activities [62]. Oleuropein exhibited cytotoxic activity against six tumoral cell lines, MCF-7, HepG2, Caco-2, A549, MDA, and DU145 [63]. Furthermore, Goldsmith and collaborators [64] assessed the anti-pancreatic cancer potential of oleuropein and its metabolite hydroxytyrosol on pancreatic cancer cells **and non-tumorigenic** pancreas cells inducing apoptosis in tumor cells, and displaying a protective effect **on non-tumorigenic** cells. The authors argued that this selectivity to the sensitivity of cancer cells is due to ROS generation. In fact, many human cancer cell types exist in a highly oxidative state compared to their normal tissues and, therefore, the selective activity of oleuropein on tumor cells could be due to their increased sensitivity towards ROS [64].

Moreover, it has been previously shown that oleuropein inhibits cell proliferation on MCF7 and HepG2 by decreasing the gene expression involved in metastasis and invasion of tumoral cells [65, 66]. Concerning ligstrosidic acid no data are available on biological activities and therefore no conclusion can be drawn.

In stem bark extracts, only FB5 showed significant SI values with 2.52 for HepG2 and 2.90 for MCF-7 while no selectivity was observed in the native crude extract FB1. Indeed, FB5 has the highest amount of polyphenol among all extract, moreover a net difference in the intensity peak of ligstroside and calceolarioside B in FB5 suggests that it could be involved in its antiproliferative activity. There is no extensive data about studies testing the biological activity of ligstroside and calceolarioside B. Principally, ligstroside aglycon was checked about possible implication against two proteins involved in breast cancer, with significant results even at low doses [67, 68].

However, it cannot be excluded that some minor components identified in the different extracts such as rutin, isoquercitrin, verbascoside and its isomer isoverbascoside may also exert pharmacological effects and therefore play a crucial role in the proprieties exerted in this study [68, 69].

## Conclusion

In conclusion, the results of the present study showed that F. angustifolia Vahl. leaves and stem bark extracts alone had no mutagenic effect on the tested strains, either in the presence or absence of metabolic activation. Moreover, significant antimutagenic activity of these extracts against several mutagens, indicates that F. angustifolia Vahl. leaves and stem bark extracts may directly protect DNA damage from mutagens, these in vitro test results would raise the question of its relation to efficacy under in vivo conditions in which factors such as bioavailability and metabolism must be taken into account.

More studies need to be carried out to assess the antimutagenic mechanisms of isolated phytochemical components of leaves and stem bark extracts of *F*. *angustifolia* Vahl. In addition to this, the antimutagenic activity of these extracts should be tested on *in vivo* model.

The strong cytotoxic activity and selectivity of FL1, FL5 and FB5 extracts against HepG2 and MCF-7 could be an indication on the potentiality of these extracts to be further screened for antiproliferative or cytotoxic activities against a number of cancer cell lines. It could be further studied for inhibitory effects on the invasion of cells at a non-lethal concentration.

## Supporting information

S1 Fig Direct mutagenic activity. Mutagenic activity of *F*. *angustifolia* Vahl. extracts (1000 μg/mL) in Ames test in the absence of the exogenous metabolic activation system (S9).
(DOCX)

S2 Fig Indirect mutagenic activity. Indirect mutagenic activity of F. angustifolia Vahl. extracts (1000 μg/mL) in Ames test in the presence of the exogenous metabolic activation system (S9).
(DOCX)

S1 Table Genotoxicity. Genotoxicity of different extracts of *F*. *angustifolia* Vahl. (1000 μg/mL) in the absence and in presence of the exogenous metabolic activation system (S9).
(DOCX)
