# Supplementary material for: Antimutagenic, antigenotoxic and antiproliferative activities of Fraxinus angustifolia Vahl. leaves and stem bark extracts and their phytochemical composition
Source: PLoS One. 2020 Apr 16;15(4):e0230690. doi: 10.1371/journal.pone.0230690 (PMC7161964; doi:10.1371/journal.pone.0230690)
Supplement: S1 Fig — Mutagenic activity of F. angustifolia Vahl. extracts (1000 μg/mL) in Ames test in the absence of the exogenous metabolic activation system (S9). (DOCX) [file pone.0230690.s001.docx]

***Fraxinus angustifolia* Vahl.**

Mutagenicity without S9

**TA100**

**TA98**

**Figure S1. Direct mutagenic activity.**

Mutagenic activity of *F. angustifolia* Vahl. extracts (1000 µg/mL) in Ames test in the absence of the exogenous metabolic activation system (S9).

Data presented as mutagenic ratio (MR) are the mean ± standard deviation (SD) of three separate experiments. Significant difference for ***p < 0.0001 (Dunnett’s test) was calculated comparing extracts to standard genotoxins: 2-NIT (2.5; 5; 10 µg/mL) and NaN3(SOD) (5; 10;20 µg/mL) used respectively for TA98 and TA100.

**L** *F.angustifolia* Vahl. leaves; **B** *F.angustifolia* Vahl. stem bark;

**1** EtOH; **2** Org/EtOAc ; **3** AQ/EtOAc ; **4** Org/CHCl_3_; **5** AQ/CHCl_3_
