# Supplementary material for: Antimutagenic, antigenotoxic and antiproliferative activities of Fraxinus angustifolia Vahl. leaves and stem bark extracts and their phytochemical composition
Source: PLoS One. 2020 Apr 16;15(4):e0230690. doi: 10.1371/journal.pone.0230690 (PMC7161964; doi:10.1371/journal.pone.0230690)
Supplement: S1 Table — Genotoxicity of different extracts of F. angustifolia Vahl. (1000 μg/mL) in the absence and in presence of the exogenous metabolic activation system (S9). (DOCX) [file pone.0230690.s003.docx]

**Table S1. Genotoxicity.**

Genotoxicity of different extracts of *F. angustifolia* Vahl*.* (1000 µg/mL) in the absence and in presence of the exogenous metabolic activation system (S9).

| Samples | IR (mean ± SD) | |
| --- | --- | --- |
|  | -S9 | +S9 |
| 4-NQO | **3.52±0.96** | - |
| 2-AA | - | **3.93±0.89** |
| FL1 | 1.10±0.45^***^ | 0.98±0.04^***^ |
| FL2 | 0.86±0.29^***^ | 0.98±0.06^***^ |
| FL3 | 0.84±0.44 ^***^ | 0.88±0.23^***^ |
| FL4 | 1.01±0.54 ^***^ | 0.86±0.04^***^ |
| FL5 | 1.16±0.26 ^***^ | 1.10±0.14^***^ |
| FB1 | 1.10±043 ^***^ | 0.94±0.07^***^ |
| FB2 | 1.03±0.31^***^ | 0.93±0.01^***^ |
| FB3 | 0.99±0.35 ^***^ | 1.12±0.09^***^ |
| FB4 | 1.031±0.15^***^ | 0.89±0.01^***^ |
| FB5 | 0.95±0.27 ^***^ | 1.19±0.15^***^ |

Data presented as induction ratios (IRs) are the mean ± standard deviation (SD) of three separate experiments. Significant difference for ^***^p < 0.001 (*Dunnett’s test*) was calculated comparing extracts to standard genotoxins: 4-NQO (0.05 µg/mL) and 2-AA (0.2 µg/mL) used in absence or in presence of S9, respectively.

**FL** *F.angustifolia* Vahl. leaves; **FB** *F.angustifolia* Vahl*.* stem bark;

**1** Ethanolic; **2** Organic/Ethyl Acetat ; **3** Aqueous/ Ethyl Acetat ;

**4** Organic/Chloroform ; **5** Aqueous/ Chloroform
